# Convalescent plasma therapy for COVID-19: a tried-and-true old strategy?

**DOI:** 10.1038/s41392-020-00310-8

**Published:** 2020-09-15

**Authors:** Yongzhi Xi

**Affiliations:** Department of Immunology and National Center for Biomedicine Analysis, Fifth Medical Center of Chinese PLA General Hospital, No. 8, Dongda Ave, Fengtai District, 100071 Beijing, P.R. China

**Keywords:** Infectious diseases, Immunotherapy

The world is currently facing a highly contagious and fatal pandemic caused by SARS-CoV-2, which is also the first pandemic caused by a coronavirus yet. As of 25 August 2020, the number of confirmed COVID-19 patients has exceeded 23,518,343 worldwide, with a death toll of over 810,492.^1^ To date, the greatest challenge we face is the incomplete knowledge of both SARS-CoV-2 and COVID-19, resulting in a lack of effective drugs and adequate treatment strategies. Despite various treatment options for critical COVID-19 patients, including extracorporeal membrane oxygenation, ventilation, tracheal intubation, circulatory support, and non-specific antiviral drugs, clinical outcomes are unsatisfactory.^2^ In particular, some mild or common COVID-19 patients experience sudden and rapid deterioration with the onset of fatal cytokine storm syndrome which cannot be treated by any conventional therapies. Therefore, from 2 February 2020 to 4 March 2020, the China National Health Commission has issued successively the 1st–7th editions of the *Official Guidance of Diagnosis and Management for COVID-19* and formulated preliminary guidelines for the clinical application of convalescent plasma therapy (CPT) for critical COVID-19 patients.^3^ However, the first edition of the official *COVID-19 Treatment Guidelines* recently published by National Health Institute clearly states that there is not sufficient clinical evidence to recommend using or not using CPT for treating critical COVID-19 patients. This stark contrast shows the lack of consensus between China and USA regarding the use of CPT for treating critical COVID-19 patients.^4^

The China National Biotec Group, under Sinopharm, co-operated with several large designated hospitals in China and initiated the use of CPT for critical COVID-19 patients. High titers of neutralizing antibodies against SARS-CoV-2 were prepared from convalescent plasma obtained from COVID-19 survivors and used to experimentally treat several critical COVID-19 patients.^5^ Within 3–12 days after receiving CPT, body temperature normalized, Sequential Organ Failure Assessment score and viral load decreased, the oxygen saturation increased, and clinical signs and symptoms of acute respiratory distress syndrome improved. As the incubation period of SARS-CoV-2 infection is 5.2–14 days, CPT should be administered within 7–14 days of infection to critical COVID-19 patients with low titers of anti-SARS-CoV-2-IgG antibodies. Previous experience has shown that CPT’s efficacy is better if used at the early stages of infection, and it is the most effective when administered before the patient produces a significant amount of IgG antibodies. During this period, the passive transfusion of high-titer and high-affinity IgG antibodies can neutralize the virus, improve the humoral immune response, reduce repeated stimulation of the immune system by killer T cells, prevent cytokine storm, shorten the disease course, and prevent the progression of infection from mild to critical. Indeed, a study in Hong Kong also indicated that, when administered within 2 weeks of disease onset, CPT reduces the durations of fever and hospital stay and significantly decreases the mortality rate in severe acute respiratory syndrome (SARS) patients.^6^

As far as CPT mechanisms are concerned, it should be particularly emphasized that so far there are several other possible direct and indirect humoral and cellular immune mechanisms by which convalescent plasma acts against virus besides neutralization.^7–9^ The direct mechanisms mainly include complement-dependent cytotoxicity, antibody-dependent cellular phagocytosis, and antibody-dependent cell-mediated cytotoxicity, by which convalescent plasma can eliminate infected cells displaying viral antigens at their surface in addition to complement-mediated inactivation of viral particles, and/or their phagocytosis. The indirect mechanisms or endogenous protective immunity (vaccine-like effects) mainly include the induction of the endogenous antiviral immune responses by convalescent plasma, such as the formation of immune complexes by convalescent plasma with virions and/or infected cells, by which convalescent plasma can enhance antiviral cytotoxic T lymphocyte responses through FcγR-mediated binding to dendritic cells, inhibit Treg expansion and immunosuppressive activity, and induce neutrophils antiviral effects.

Although CPT is well established and clinically feasible, it still faces great challenges. As is well known, von Behring and Kitasato first discovered antisera against *Clostridium tetani* and *Corynebacterium diphtheriae* infections and saved thousands of children with diphtheria in 1890. For this rudimentary CPT method, von Behring^10^ was awarded the first Nobel Prize in Physiology/Medicine in 1901. In the first half of the twentieth century, CPT was usually used against various human bacterial infections. CPT was first used for viral infection during the Spanish influenza in 1918, when a small number of critical patients were experimentally treated with convalescent plasma.^11^ Since then, CPT has been often used to treat highly infectious viral diseases in emergency conditions, including COVID-19, Ebola, Middle East respiratory syndrome (MERS), and SARS.^5,6,12,13^ However, individual cases or non-randomized, observational, experimental treatments in small samples make up the bulk of the studies of CPT; this makes it difficult to draw scientifically sound conclusions about its usefulness. As everyone knows that modern medicine is built on such rigorous, strict, and meticulous scientific system. Dawkins R, one of the most famous atheists in the world, fellow of the Royal Society in UK, and a Professor at the Oxford University, believes that the scientific concept that can best improve everyone’s cognitive ability is the “double-blinded controlled trial”. Thus, the gold standard to prove CPT’s efficacy is to conduct a set of large-scale, double-blind, randomized controlled clinical trials (RCT), which will provide scientifically accurate data and reliable conclusions.^14^ Therefore, taking the opportunity of this SARS-CoV-2 outbreak, with critical COVID-19 patients, one must attempt to reduce CPT’s shortcomings. Meanwhile, we also need to realize that the bottleneck of RCT application and feasibility in critical patients receiving CPT during infectious diseases outbreak: (1) critical patients with infectious diseases usually have a short period of disease, rapid progression, and high mortality, so that it is difficult to implement RCT step by step; (2) critical patients’ risk factors and clinical center’s specific supportive care also will influence RCT application and feasibility; and (3) in such a limited time, it is extremely difficult to collect standard convalescent plasma with high-titer and high-affinity IgG antibodies. Beyond these, in order to greatly improve CPT efficacy for major refractory infectious diseases in the future, we should also strengthen the relevant basic research on CPT: (1) to in-depth study the unknown mechanisms by which convalescent plasma acts in addition to those known above; (2) to compare the curative effect between conventional convalescent plasma and polyclonal or monoclonal antibodies or pooled human immunoglobulin Ig prepared from convalescent blood, plasma, or serum; and (3) to explore and discover the synergistic or additive effect of the known drugs with CPT.

**Fig. 1:**
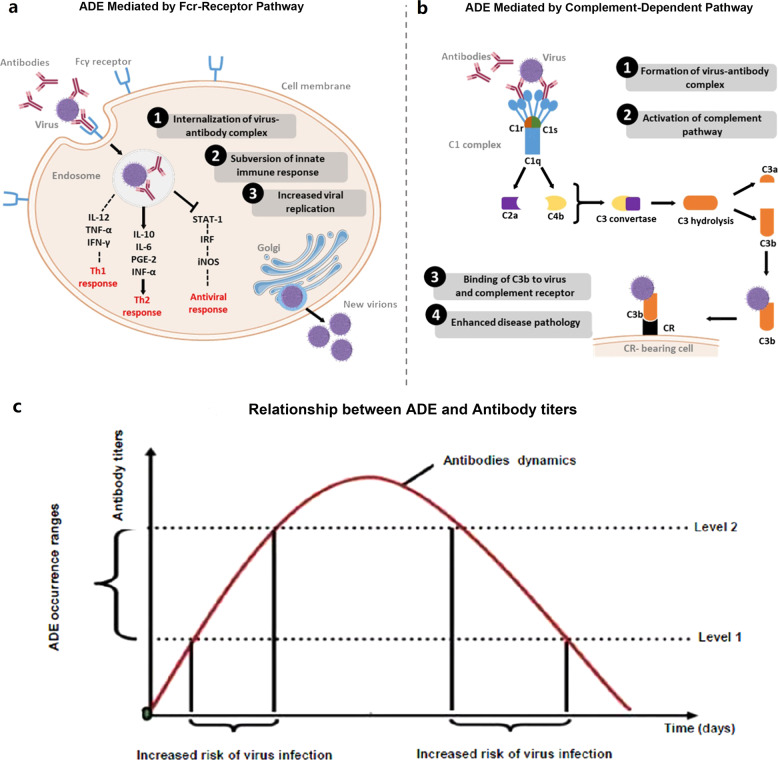
The immune mechanisms of ADE mediated by viral infections. **a–c** Mechanisms underlying the antibody-dependent enhancement (ADE) of viral infections mediated by the Fcγ receptor and complement-dependent pathways, and the relationship between ADE occurrence and antibody titer. Figure partially adapted from ref. ^15^

Another major challenge for CPT is the antibody-dependent enhancement (ADE) of viral infection mediated by preexisting enhancing, non-neutralizing, or sub-neutralizing levels of antibodies from the convalescent plasma administered. It has been confirmed that these polyclonal or monoclonal antibodies can induce ADE in virally infected humans and animals via mechanisms involving the Fcγ receptor and complement-dependent pathways (Fig. 1a–c).^15^ The binding of virions to this class of antibodies could promote cellular entry, enhance viral replication, cause persistent viremia, and exacerbate illness. To date, ADE has been reported in more than 40 families of viruses, including human coronaviruses SARS-CoV-1 and MERS-CoV.^16,17^ As expected, the latest study has shown that anti-SARS-CoV-2 antibody can also cause ADE in COVID-19 experiments.^18,19^ During SARS-CoV infection, different types of antibodies against spike glycoproteins S1–S4, envelope glycoprotein E, membrane glycoprotein M, and nucleocapsid N are produced, representing a mixture of neutralizing/non-neutralizing and enhancing/non-enhancing antibodies. In particular, some kinds of spike-specific-IgG antibodies have been characterized as being more of the enhancing than the neutralizing type, thereby promoting SARS-CoV infection in human immune cells via canonical Fcγ-receptor II and non-canonical angiotensin I-converting enzyme 2 (ACE2) pathways.^17^ Studies on several animal models have shown that some kinds of spike-specific-IgG antibodies cause severe acute diffuse pulmonary alveolar damage by subverting the immune response, including reducing Th1-cytokines, increasing Th2-cytokines, and inhibiting the STAT pathway and monocyte/macrophage accumulation in the lungs. Therefore, it is necessary to identify the convalescent plasma that has an ADE effect using cell lines expressing either Fcγ- or complement-, and even ACE2- receptors before clinical application in critical COVID-19 patients.^17^ It is essential to confirm that CPT application does not induce ADE of SARS-CoV-2 infection.

Human plasma usually contains 10^7^ to 10^8^ different types of antibodies, of which only 40 or more are neutralizing antibodies. In critical patients with emerging infectious diseases, CPT’s efficacy is closely related to the types and titers of specific neutralizing antibodies in the convalescent plasma administered.^6,20^ Therefore, it is important to determine the types of specific neutralizing antibodies and the dynamics of antibody titers in the convalescent plasma. For example, SARS-CoV-1 patients produce specific antibodies not only against the predominant structural proteins but also against several known and unknown protein antigens of the virus. The production, concentration, and dynamics of each of these antibodies show distinct trends. Specific antibodies against proteins S, N, 3a, and 9b, but not against proteins E, M, 3b, and 7a, have been detected in the sera of SARS-CoV-2 patients who have recovered.^17^ The decline in the antibodies against S protein, N protein, and the receptor-binding region (RBD) of S protein in the convalescent plasma shows different trends. Usually, anti-N antibodies decreased rapidly from the third month to the 12th month and then decreased slowly while staying at a low level slightly above the threshold value; anti-S antibodies and anti-RBD antibodies IgG remained at a high level from the third month to the 36th month and then slowly declined. Therefore, to collect high quality, clinically efficacious convalescent plasma, it is pertinent to consider the dynamics of different antibodies it contains. At present, we can use pseudoviruses neutralization test to identified neutralizing antibody against SARS-CoV-2.^21^ The identification of enhancing antibody can be done by cell lines expressing Fcγ- and complement-receptors.^15^ The indirect ELISA test can be used to identified binding antibody.

Undoubtedly, CPT has many known and unknown risk factors, in addition to ADE, such as the differences in the types and titers of specific and non-specific antibodies in convalescent plasma obtained from different patients and the presence of other potential pathogens in virus-inactivated plasma. Therefore, while conducting large-scale clinical trials of CPT in critical COVID-19 patients, the guidelines for convalescent plasma collection, as specified in the blood donation laws and pharmacopoeias, must be strictly followed, and standard procedures must be maintained during the entire process. In addition to routine screening for pathogens, convalescent plasma must be tested rigorously for pathogens of respiratory, gastrointestinal, and urogenital diseases. Virus inactivation must be done meticulously to ensure safety in the clinical application of CPT.^22^ Besides, it must be soberly realized that the large-scale collection of convalescent plasma within the limited time of 2–4 weeks will present a plethora of practical obstacles and difficulties, including unwilling donors and failure to meet donation eligibility criteria, etc. As indicated in a large-scale double-blinded randomized controlled study involving 9101 H1N1 patients who recovered, only 7.5% individuals could be successfully recruited for convalescent plasma or whole blood donation.^23^

As it is difficult to develop new effective antiviral drugs in a short time, in the face of successive major outbreaks of emerging infectious diseases, repurposing existing drugs and therapies can be a viable option. In an emergency, the long-established CPT will likely emerge as the treatment of choice for critical patients; however, it must overcome the stated unresolved challenges.
